# *Aspergillus flavus* NRRL 35739, a Poor Biocontrol Agent, May Have Increased Relative Expression of Stress Response Genes

**DOI:** 10.3390/jof5020053

**Published:** 2019-06-20

**Authors:** Kayla K. Pennerman, Guohua Yin, Joan W. Bennett, Sui-Sheng T. Hua

**Affiliations:** 1 Department of Plant Biology, Rutgers University, The State University of New Jersey, New Brunswick, NJ 08901, USA; kkpennerman@gmail.com (K.K.P.); guohuayin1997@gmail.com (G.Y.); profmycogirl@yahoo.com (J.W.B.); 2 Foodborne Toxin Detection and Prevention Research, United States Department of Agriculture, Agricultural Research Service, Albany, CA 94710, USA

**Keywords:** *Aspergillus flavus*, aflatoxin biocontrol, comparative transcriptomics, abiotic stress

## Abstract

Biocontrol of the mycotoxin aflatoxin utilizes non-aflatoxigenic strains of *Aspergillus flavus*, which have variable success rates as biocontrol agents. One non-aflatoxigenic strain, NRRL 35739, is a notably poor biocontrol agent. Its growth in artificial cultures and on peanut kernels was found to be slower than that of two aflatoxigenic strains, and NRRL 35739 exhibited less sporulation when grown on peanuts. The non-aflatoxigenic strain did not greatly prevent aflatoxin accumulation. Comparison of the transcriptomes of aflatoxigenic and non-aflatoxigenic *A. flavus* strains AF36, AF70, NRRL 3357, NRRL 35739, and WRRL 1519 indicated that strain NRRL 35739 had increased relative expression of six heat shock and stress response proteins, with the genes having relative read counts in NRRL 35739 that were 25 to 410 times more than in the other four strains. These preliminary findings tracked with current thought that aflatoxin biocontrol efficacy is related to the ability of a non-aflatoxigenic strain to out-compete aflatoxigenic ones. The slower growth of NRRL 35739 might be due to lower stress tolerance or overexpression of stress response(s). Further study of NRRL 35739 is needed to refine our understanding of the genetic basis of competitiveness among *A. flavus* strains.

## 1. Introduction

Aflatoxins are highly carcinogenic mycotoxins that present major worldwide economic and health challenges [1,2,3,4]. The filamentous fungus *Aspergillus flavus* is the most common cause of aflatoxin contamination of foodstuffs and animal feeds. Field application of a non-aflatoxigenic strain of *A. flavus* is often used as a biocontrol strategy to reduce aflatoxin accumulation [5,6]. These non-aflatoxigenic strains lack the ability to produce aflatoxins due to disruptions in the aflatoxin biosynthesis gene cluster [7,8,9]. Before a non-aflatoxigenic strain is used as a biocontrol agent, it is helpful to verify that the strain does not have or express genes required for aflatoxin production. Most non-aflatoxigenic strains have deletions that fall into one of eight patterns, named A to H, which can be easily determined with traditional PCR or genomic sequencing [7]. For example, WRRL 1519 exhibits pattern E deletion, lacking the first half of the gene cluster (genes *aflF* to *aflE*) [10]. NRRL 21882 (Afla-Guard) has the largest deletion pattern, H [7]. However, not every non-aflatoxigenic strain shares one of these deletion patterns. For example, the commercial biocontrol agents NRRL 30797 and NRRL 18543 have just a few substitutions and/or deletions in the aflatoxin biosynthesis gene cluster [7,11]. Further, a new Korean strain of *Aspergillus oryzae* that is highly effective in reducing aflatoxin contamination has deletions throughout the cluster [12].

Non-aflatoxigenic *A. flavus* strains vary in how effective they are in preventing aflatoxin accumulation when grown in the presence of toxigenic strains. Commercially available strains tend to be strong biocontrol agents, but other, less-effective, strains also exist [5,13,14]. Most reports of new non-aflatoxigenic strains skew towards identifying and studying the biocontrol agents with greater potential that many researchers believe prevent aflatoxin accumulation by out-competing aflatoxigenic strains [15,16,17]. Assayed visually, 80% of the fluorescently labeled aflatoxigenic *A. flavus* AF70 is suppressed when grown in corn kernels and co-inoculated with the non-aflatoxigenic NRRL 18543 [15]. Co-inoculation of two aflatoxigenic strains also results in lower aflatoxin yields than expected, suggesting that the inability of a given aflatoxigenic strain to create a sufficient mycelial network contributes to biocontrol effectiveness [18,19]. A similar effect is seen in apples inoculated with two strains of toxigenic *Penicilium expansum* in the same wound; the resulting levels of patulin decrease [20]. Another relevant hypothesis states that mycotoxin production is a mechanism to help alleviate oxidative stress [16,17,21,22]. Stronger biocontrol agents may be particularly good at enduring oxidative stress [16].

Omics technologies help decrypt the natural variation of these biocontrol agents while yielding abundant data with frequently ambiguous results [23,24,25,26,27]. Investigating a weak *A. flavus* biocontrol agent would help to reduce background noise in these data and help pinpoint stronger signals that contribute to biocontrol efficacy. To this end, we have investigated *A. flavus* NRRL 35739 (also known as strain NPL TX13-5), an exceptionally poor biocontrol agent with pattern E deletion [7,26]. Despite being non-aflatoxigenic, this strain does not significantly reduce aflatoxin accumulation when co-inoculated with several aflatoxigenic strains on peanut. More perplexingly, in some instances, the strain even increases toxin production [26]. It was hypothesized that NRRL 35739 is a poor biocontrol agent due to a lack of reduced expression of redox genes that could be revealed via comparative transcriptomics. However, results from the present study indicate that this strain may have a lower tolerance for normal growth conditions associated with an increased relative expression of general stress genes.

## 2. Materials and Methods

### 2.1. Fungal Strains, Aflatoxin Biocontrol, and Abiotic Stress Assays

Freeze-dried samples of strains NRRL 3357 (aflatoxigenic), NRRL 6513 (aflatoxigenic), and NRRL 35739 (non-aflatoxigenic) were received from the NRRL Culture Collection (https://nrrl.ncaur.usda.gov/). Spores of the fungi grown on potato dextrose agar (PDA; Difco) for one week in the dark at 32 °C were gently harvested and diluted to 10^4^, 10^6^, or 10^7^ spores/mL in a sterile aqueous solution of 10% *v/v* glycerol and 1% *v/v* Tween 80 (spore suspension liquid). Spores were stored at −20 °C until use. Before each experiment, spores for each strain were inoculated and grown on three plates of PDA at 32 °C for one week. The new spores were gently harvested in 10 mL of spore suspension liquid and filtered through eight layers of sterile cheesecloth. Collected spores were stored at 4 °C for a maximum of two weeks.

To determine if NRRL 35739 was capable of decreasing aflatoxin accumulation, 100 µL of 10^4^ NRRL 35739 spores/mL was co-inoculated with the same amount of either aflatoxigenic strain NRRL 3357 or NRRL 6513 in three flasks containing 50 mL of YES medium (20 g of yeast extract, 150 g of sucrose per liter). Cultures were grown at 32 °C, shaking at 150 rpm on an Innova 2000 platform shaker (New Brunswick Scientific) for six days in the dark. Aflatoxin was extracted from the spent media following a published method with some exceptions [16]. Briefly, 3 mL from each culture flask was centrifuged at 10,000× g for 5 min to pellet hyphae. Two 500 μL samples of the supernatant were each quickly mixed with 1 mL of methyl chloride and centrifuged at 10,000× g for 2 min. The organic bottom layers were transferred to a glass vial and allowed to evaporate. Dried extracts were gently dissolved in 100 µL methyl chloride; 30 µL of the solutions were loaded onto 4 × 8 cm TLC silica gel 60 F254 plates (Millipore Sigma) along with 15 drops of an aflatoxin mix with an unspecified concentration in 98:2 benzene:acetonitrile (Millipore Sigma). The plates were developed with 8:1 ethyl acetate:toulene. Aflatoxin fluoresced under ultraviolet light. The experiment was replicated three times.

For the abiotic stress assays, 5 µL of 10^6^ spores of each *A. flavus* strain were three-point inoculated on three solid YES media (with 15 g of bacto-agar per liter) and incubated for two days in the dark at 32 °C. After incubation, the diameters of the colonies were measured. Stresses were introduced by amending the sterile medium to contain 5 mM, 10 mM, or 15 mM hydrogen peroxide; 2%, 4%, or 8% (w/v) sodium chloride; 5%, 10%, or 15% (*v/v*) glycerol; or by growing cultures at 22 °C, 27 °C, 37 °C, or 42 °C. The experiment was replicated four times. Two-tailed Student’s *t*-tests with the α-level set to 10^−5^ were performed to find significant differences from the respective control group for each strain.

Growth on a peanut host was also tested. Raw peanuts were purchased from a local supermarket, shelled, and the seed coats were removed. Five kernels were halved, rinsed vigorously with sterile water, and submerged in sterile water or a 10^7^ *A. flavus* strain spore per milliliter suspension for 30 min. Kernels were shaken to remove excess liquid and placed on a filter paper in a Petri dish with 1 mL of sterile water. Plates were sealed with Parafilm and incubated for four days in the dark at 32 °C. The experiment was performed three times.

### 2.2. RT-PCRs and Comparative Transcriptomics

Two replicates of the *A. flavus* strains NRRL 3357, NRRL 6513, and NRRL 35739 were grown on potato dextrose broth for 10 days at 32 °C in the dark. Total RNA was extracted and converted to cDNA using the Zymo Research Quick-RNA Fungal/Bacterial MiniPrep Kit (Integrated Sciences) and the High-Capacity cDNA Reverse Transcription Kit (Thermo Fisher Scientific) as directed by the manufacturers. The RT-PCR products were stored at −20 °C. Primers targeting *GAPDH* and six putative *A. flavus* NRRL 3357 stress response genes (National Center for Biotechnology Information, NCBI, accessions AFLA_006960, AFLA_019230, AFLA_022380, AFLA_037820, AFLA_060260, AFLA_079830) were used with the DreamTaq Green PCR Master Mix (Table 1). The PCR conditions were denaturation at 95 °C for 30 s, annealing at 50 °C for 30 s, and extension at 72 °C for 1 min, cycling 25 times. Ten microliters of the PCR products were subjected to gel electrophoresis on a 2% agarose gel. PCRs were repeated once. It was assumed that the three strains expressed *GAPDH* at similar levels, and the *GAPDH* PCR products were used as an internal control for the amount of cDNA template used in PCRs.

Fifty milliliters of potato dextrose broth (Difco) in a 250 mL Erlenmeyer flask was inoculated with 50 µL of 10^4^ NRRL 35739 spores/mL and incubated for 10 days at 32 °C in the dark without shaking. Total RNA was extracted from the other culture using a Zymo Research Quick-RNA Fungal/Bacterial kit according to the manufacturer’s instructions. The extracted RNA was submitted to GENEWIZ (South Plainfield, New Jersey). After determining that the RNA sample had an RNA Integrity Number of 10.0, the sample was subjected to poly(A) selection and then sequenced on an Illumina HiSeq 2500 platform. Raw reads were uploaded to the NCBI Sequence Read Archive (SRA) database under accession PRJNA520841.

RNA-Seq reads from aflatoxigenic (AF70 and NRRL 3357) and biocontrol (NRRL 18543 and WRRL 1519) strains were retrieved from the NCBI SRA database (Table 2). Read quality was checked with FastQC version 0.11.7 [28]. All the reads were aligned to the genomes of AF70 and NRRL 3357 as these two were the best annotated *A. flavus* genomes and represented S- and L-morphotypes, which have different gene contents and organizations [29,30,31]. For this, STAR version 2.6 was used with the accompanying GFF files (NCBI assemblies GCA_000952835.1 and GCA_000006275.2) [31,32,33]. The output SAM files were converted to sorted BAM files using Samtools version 1.7 [34]. Stringtie version 1.3.5 and Python were used to generate fragments per kilobase of transcript per million mapped reads (FPKM) matrices [35,36]. For each strain, FPKMs were normalized on a scale of 0 to 100 by dividing each number by one-hundredth of the highest FPKM value for that strain. Relative FPKMs were then compared for identification of genes for which (1) the absolute difference in relative FPKM for NRRL 18543 and WRRL 1519 was within 0.5 units, and (2) the fold changes in relative expression in NRRL 35739 were at least 10-fold in difference to that in NRRL 18543 and WRRL 1519. Graphs were generated using Inkscape version 0.91 (available at: https://inkscape.org/en/) and R [37]. Annotations of genes of interest were verified using NCBI BLAST and InterProScan version 5.32 [38,39]. To identify differences in promoter regions, sequences 500 bp upstream of genes of interest were collected and aligned to one another using Clustal Omega [40].

## 3. Results

### 3.1. Strain NRRL 35739 was Poor at Biocontrol and Grew More Slowly

**Figure 1:**
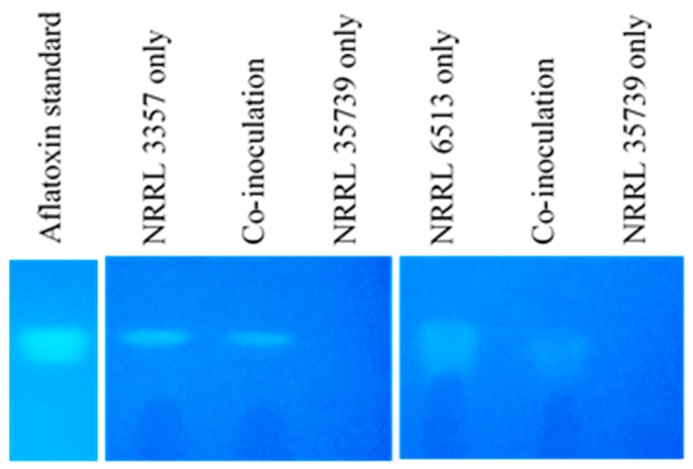
Weak biocontrol ability of *A. flavus* NRRL 35739. The strain was tested against NRRL 3357 (middle) and NRRL 6513 (right). Co-inoculation of an aflatoxigenic strain with NRRL 35739 did not greatly affect aflatoxin accumulation in the media. The retention factors of the aflatoxin extracts were similar to that of the aflatoxin standard (left). Images are representative of three replicates.

Strains NRRL 3357 and NRRL 6513 produced aflatoxin that was detectable regardless of co-inoculation with NRRL 35739 (Figure 1). For both aflatoxigenic strains, co-inoculation with the non-aflatoxigenic strain did not visibly affect detected toxin accumulation or caused a slight, unquantified decrease in aflatoxin fluorescence. Media in which only NRRL 35739 was inoculated did not have aflatoxin.

**Figure 2:**
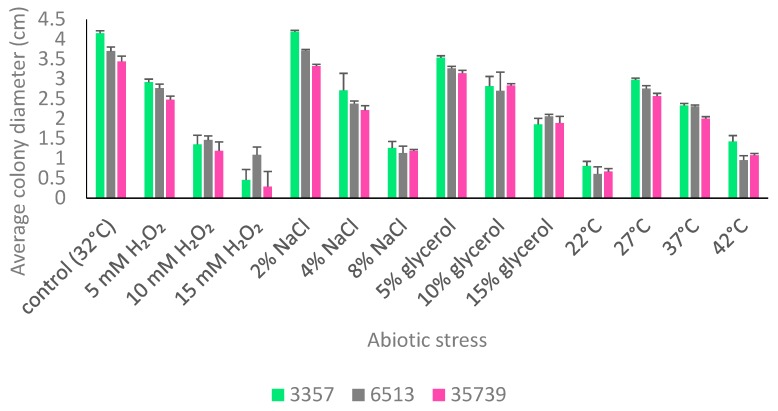
Abiotic stress tolerance of *A. flavus* NRRL 3357, NRRL 6513, and NRRL 35739. Cultures were grown on solid YES media amended with the indicated stress agents. Colony diameters were measured after two days. Error bars represent one standard deviation above the mean of four replicates.

**Figure 3:**
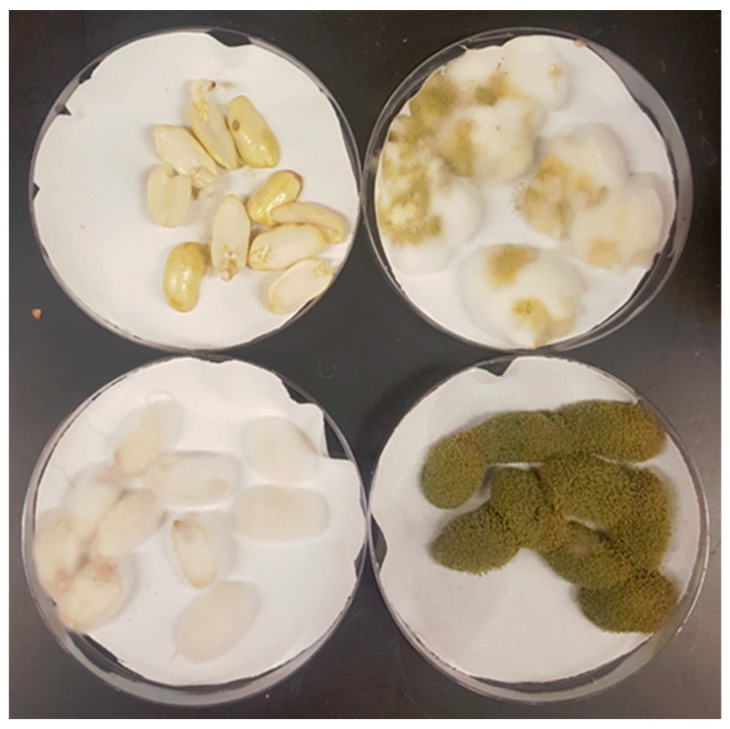
Growth of three *A. flavus* strains on raw peanuts. After four days, growths of NRRL 35739 on the hosts were less thick than the fluffy or heavily sporulating growths of aflatoxigenic strains NRRL 3357 and NRRL 6513. Clockwise from the top left: negative control, NRRL 3357, NRRL 6513, and NRRL 35739. The image is representative of three replicates.

The abilities of the three strains to withstand oxidative, salinity, dehydration, cold, and heat stresses were tested. All of the stressors significantly (*p* < 10^−5^) reduced growth except for 2% sodium chloride (Figure 2). NRRL 35739 generally had smaller colony sizes than NRRL 3357 and NRRL 6513 for most treatment groups. However, the fold changes by which the growths of the strains were affected by each stress were largely consistent. Compared to the other two strains, NRRL 6513 was slightly more tolerant of 15 mM hydrogen peroxide and slightly less tolerant of incubation at 42 °C. NRRL 35739 also formed visibly less thick hyphal coatings on raw peanuts (Figure 3). NRRL 6513, which was generally intermediate in growth between the other two strains on control and amended YES media, sporulated more heavily than the other two strains when grown on peanuts, but not on the solid YES medium. NRRL 35739 sporulated on solid artificial medium.

### 3.2. RNA-Seq Data Revealed Differences in the Transcriptomes of A. flavus Strains

**Figure 4:**
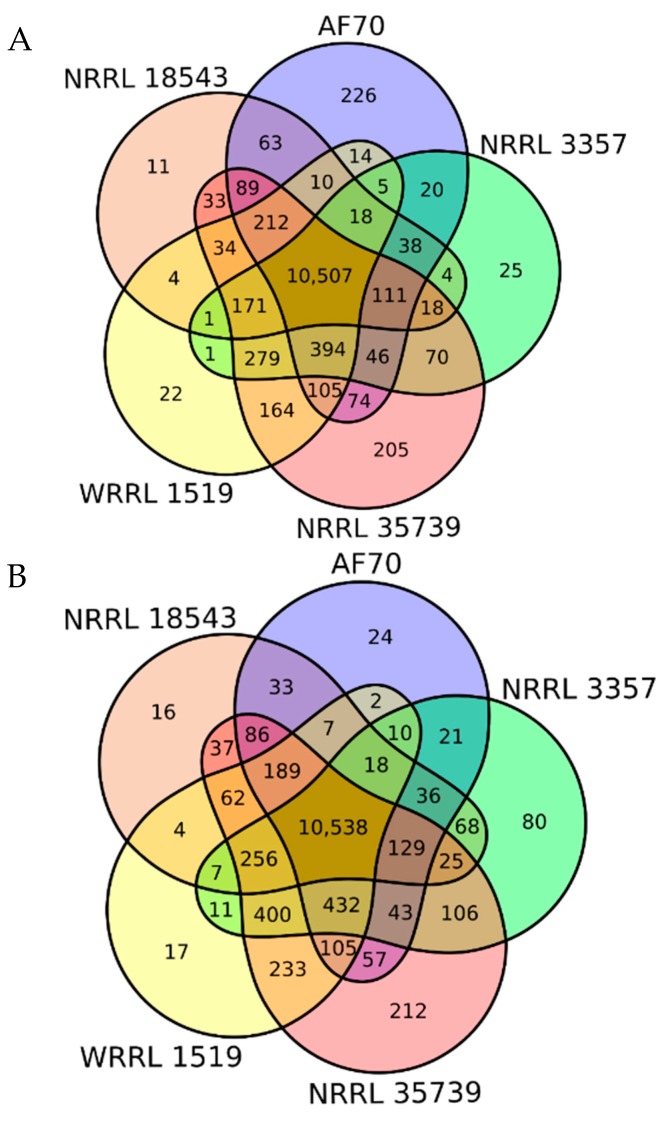
Venn diagrams of commonly expressed genes for selected *A. flavus* strains. Genes that had FPKMs greater than 0 were counted according to RNA sequence read alignment to the genomes of (**A**) AF70 or (**B**) NRRL 3357. A total of 222 AF70 genes and 221 NRRL 3357 genes were not expressed by any of the five strains and are not represented in the figure. The total number of annotated genes in the AF70 and NRRL 3357 genomes were 13,196 and 13,485, respectively.

A yield of 283,801,877 high-quality (88.24% ≥ Q30 with a mean quality score of 37.2) 2 × 150 bp paired-end sequencing reads without adapter sequences of the NRRL 35739 transcriptome was obtained. The majority of RNA-Seq reads from strains AF70, NRRL 18543, NRRL 3357, NRRL 35739, and WRRL 1519 were aligned to the genomes of AF70 and NRRL 3357 (Table 3). Generally, the reads better aligned to the genome of AF70 than to that of NRRL 3357 for unknown reasons. However, most of the expressed genes were commonly expressed by all of the strains, regardless of which genome was used for read alignment (Figure 4).

Following read alignment to either genome, genes with FPKMs greater than 0 were considered to be expressed by a particular strain. Genes that were uniquely expressed by the non-aflatoxigenic strains NRRL 18543, WRRL 1519, or NRRL 35739 were annotated mostly as hypothetical proteins and had low relative expressions (relative FPKM < 0.2). Three of the few functionally annotated genes expressed only by NRRL 18543 or WRRL 1519 were membrane transporters, oxidoreductases, and polyketide synthetases. None of the genes exclusively expressed by both had functional annotations. Among the genes that were uniquely expressed by NRRL 35739, the genes were mostly hypothetical, transporters/permeases, or dehydrogenases. The genes expressed only by AF70 and NRRL 3357 included genes in the aflatoxin biosynthesis gene cluster, as well as a glucose dehydrogenase and a multidrug resistance protein.

As expected, the aflatoxigenic strains expressed most of the aflatoxin biosynthesis genes, regardless of to which genome the RNA reads were aligned, although AF70 did not detectably express *aflLa*, *aflI*, or *aflQ*, and NRRL 3357 did not express *aflV*. In alignment AF70 (RNA reads aligned to the genome of AF70), NRRL 3357 additionally did not express *aflLa*. The non-aflatoxigenic strains also expressed some of the genes that were present in their respective genomes. For both alignments, strain NRRL 18543 expressed *aflT* to *aflS*, *aflMa*, and *aflNa* and *aflX* to *aflYe*. WRRL 1519 expressed *aflN* to *aflL*, *aflQ*, and *aflW* and *aflYb* to *aflYe*. NRRL 35739 expressed *aflM* to *aflG*, *aflP* to *aflW*, and *aflYa* to *aflYe*; it was the only one of the non-aflatoxigenic strains to express *aflM*, *aflP*, *aflK*, and *aflV*. According to alignment AF70, NRRL 18543 expressed *aflF* and *aflU*, and NRRL 35739 expressed *aflI*, *aflO*, *aflX*, and *aflY*. NRRL 35739 expressed all aflatoxin biosynthesis genes that NRRL 18543 and WRRL 1519 did.

For both alignments, the most highly expressed annotated genes for AF70 and NRRL 18543 were similar: a glutamine synthetase, three conidiation genes, an extracellular 3-ketosteroid 1-dehydrogenase, and a heat shock protein. NRRL 3357 highly expressed two allergens, a pyruvate decarboxylase, a translation elongation factor, an ADP/ATP carrier protein, and a manganese superoxide dismutase. For WRRL 1519, the most highly expressed genes included a glyceraldehyde 3-phosphate dehydrogenase, a 60S ribosomal protein, and the same manganese superoxide dismutase and ADP/ATP carrier protein as NRRL 3357. The top genes expressed by NRRL 35739 included a heat shock protein, one of the two allergens highly expressed by NRRL 3357, a CsbD-like general stress response protein, a translation elongation factor, and a high expression lethality protein.

### 3.3. NRRL 35739 had Higher Relative Expression of Six Stress-Related Genes

Relative gene expressions were used to identify genes that had large differences between the strong biocontrol strains and NRRL 35739. A total of 26 genes from the AF70 and NRRL 3357 alignments had relative gene expressions that were similar between strains NRRL 18543 and WRRL 1519, but different for NRRL 35739 (Table 4 and Table 5). Coincidentally, all the identified genes had higher relative expression in NRRL 35739. Seven of the genes (AFLA70_502g000700, AFLA70_560g000680, AFLA70_73g004291, AFLA70_89g003151, AFLA_019230, AFLA_037820, and AFLA_060260) were involved in stress response.

The most highly expressed functionally annotated genes of interest in either alignment were the heat shock protein homologs AFLA70_560g000680 and AFLA_060260. The only other gene with higher relative expression in the NRRL 3357 alignment of NRRL 35739 reads was the hypothetical gene AFLA_099070. The relative transcriptome landscapes of the strains were visualized (Figure S1).

**Figure 5:**
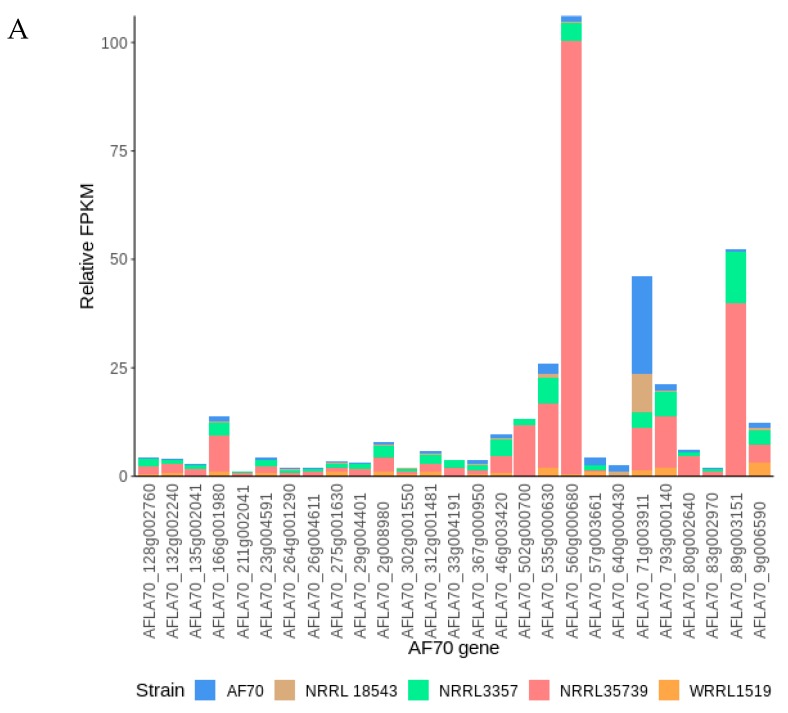

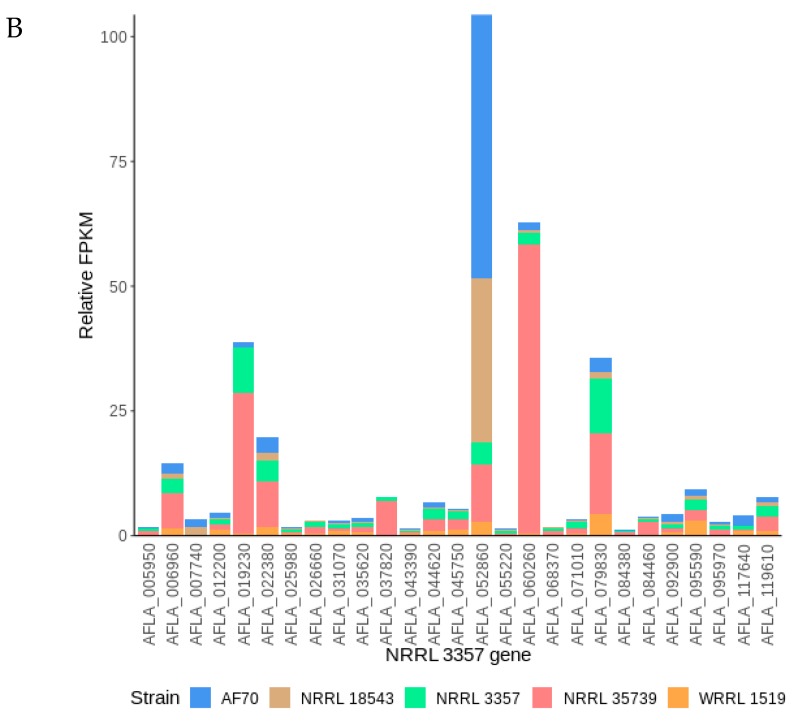
Relative expressions of heat shock and other stress-related genes in five *A. flavus* strains. Graphs were based on (**A**) AF70 and (**B**) NRRL 3357 alignments. Genes with total relative FPKMs less than 1 were excluded. Gene functional annotations are described in Table 6.

**Figure 6:**
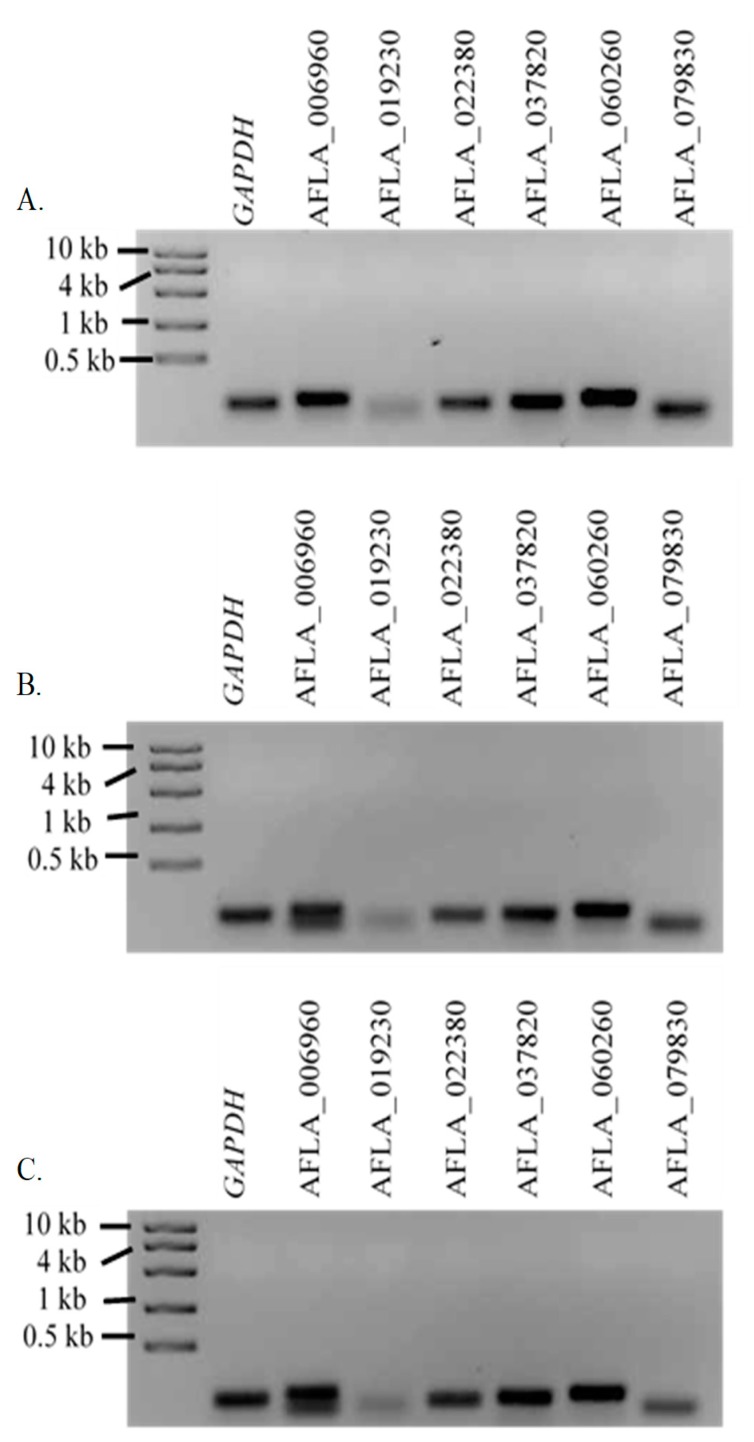
Transcript accumulation of selected genes by RT-PCR. *GAPDH* served as a control for template loading. Intensities of bands were visually compared among *A. flavus* strains (**A**) NRRL 3357, (**B**) NRRL 6513, and (**C**) NRRL 35739.

The higher relative expression of two heat shock proteins in both alignments prompted a closer look at the relative expressions of other genes annotated as being involved in heat shock and stress responses (Table 6 and Figure 5). One pair of homologous AF70 and NRRL 3357 heat shock proteins (AFLA70_71g003911 and AFLA_052860) were relatively more expressed in AF70 and NRRL 18543. Higher relative expressions were observed for six other pairs of homologous genes (AFLA70_166g001980, AFLA70_502g000700, AFLA70_535g000630, AFLA70_560g000680, AFLA70_793g000140, AFLA70_89g003151, AFLA_006960, AFLA_019230, AFLA_022380, AFLA_037820, AFLA_060260, and AFLA_079830), NRRL 35739. However, these data were not confirmed by RT-PCR (Figure 6). Comparisons of the upstream regions of the genes of interest did not reveal any differences among them.

## 4. Discussion

It was hypothesized that the poor biocontrol strain *A. flavus* NRRL 35739 would have a lower tolerance for oxidative stress than aflatoxigenic strains as well as reduced expression of redox genes compared to the stronger biocontrol agents. However, NRRL 35739 generally grew slower regardless of the tested abiotic stresses. From preliminary comparative transcriptomics with data derived from other studies, it seemed that the redox genes mostly were expressed at similar relative levels as aflatoxigenic and strong biocontrol strains. Instead, genes for heat shock proteins (AFLA70_166g001980, AFLA70_502g000700, AFLA70_535g000630, AFLA70_560g000680, AFLA_006960, AFLA_022380, AFLA_037820, and AFLA_060260), a CsbD-like protein (AFLA70_89g003151/AFLA_019230), and a rare cold inducible (RCI) stress response peptide (AFLA70_793g000140/AFLA_079830) appeared to be relatively more highly expressed in NRRL 35739 compared to the other four *A. flavus* strains. RT-PCRs did not confirm higher expressions of the genes of interest in NRRL 35739 than in NRRL 3357. It would be more informative to test the relative expressions of these genes between NRRL 35739 and a strong biocontrol strain, which, unfortunately, we were unable to obtain for this work.

Heat shock proteins are a ubiquitous family of proteins involved in responses to many abiotic stresses, including heat, salinity, and oxidative stresses [43,44,45,46,47]. They are named by their molecular weight in kildodaltons and often serve as molecular chaperones, helping nascent and denatured peptides fold properly or be tagged for degradation. *Aspergillus* heat shock protein 90, an antigen in allergic bronchopulmonary aspergillosis by *Aspergillus fumigatus*, promotes drug resistance and conidiation and is involved in signal transduction [48,49,50,51,52,53,54,55,56]. Hsp70 facilitates protein folding and plays a role in resistance to antifungal drugs [57,58]. Hsp42 suppresses aggregation of cytosolic proteins [59]. Hsp40 is a co-factor for Hsp70 and facilitates protein folding [44,60]. Hsp30 interacts with Hsp80 and Hsp70 to help fold polypeptides and helps conserve energy by inhibiting ATPase [61,62,63]. Heat shock proteins in the 20–30 kD range facilitate protection against heat and oxidative stresses, nuclear import of a protein kinase in human cardiac cells, and cellular motility [64,65,66,67]. Hsp12 is upregulated during heat, oxidative, and cold stresses, increasing membrane stability and causing morphological changes [61,68,69]. Homologous to Hsp12, Hsp9 is involved in passing the G2-M checkpoint of the cell cycle and is induced by heat shock, glucose deprivation, and stationary growth in the yeast *Schizosaccharomyces pombe* [70,71].

*csbD* is a gene of unknown function that is induced during stress in bacteria [72,73,74,75]. AFLA70_89g003151 and AFLA_019230 were originally described as mismatched base pair and cruciform DNA recognition proteins. However, both contain the conserved CsbD superfamily domain in the middle of the predicted protein and no other known domains. The RCI stress peptide responds to several abiotic stresses in plants and *Saccharomyces cerevisiae* and localizes to yeast membranes [76,77,78]. All genes of interest are upregulated by hydrogen peroxide and resveratrol in NRRL 3357, except for AFLA_019230 [17,79]. AFLA_060260 is additionally significantly upregulated in *A. flavus* NRRL 3357 at 37 °C compared to 28 °C in YES medium [80]. The *Aspergillus nidulans* heat shock protein genes AN2530 (Hsp20-like), AN3463 (Hsp40-like), AN4037 (hypothetical thermotolerance gene), and AN7892 (Hsp20/Hsp30-like) have increased expression under carbon starvation, while expression of an RCI peptide gene, AN2312, decreases [81]. Heat shock proteins are important for response to nutrient stress in *S*. *cerevisiae* and *Escherichia coli* [82,83,84,85,86].

It was not clear why NRRL 35739 may have had increased relative expression of stress proteins. If NRRL 35739 was reacting to heat stress, there would have been accompanying high expressions of oxidative stress responses [87,88,89]. The strain was grown at 32 °C before RNA extraction unlike the other strains, which were grown at 28–31 °C. However, the differences in temperature were minor and within a non-detrimental temperature range for *A. flavus* growth [90,91,92]. High relative expressions of an Hsp9/12 gene, AFLA70_71g003911/AFLA_052860, for strains AF70 and NRRL 18543 were also noted and likewise remain unexplained. The transcriptomic data for the strains were collected during the same experiment, so the results may have been particular to *A. flavus* strains with S-morphotype lineages or to certain experimental conditions that were not replicated in the present study.

Increased expression of stress proteins might indicate nutrient stress. Organisms in the same area compete for resources they need to grow, survive, and reproduce. The ability to be a better competitor relies on the ability to better utilize available resources and/or be better able to tolerate less than optimal conditions [93]. *A. flavus* biocontrol strains differ from most other fungal biocontrol agents in that *A. flavus* strains are used to reduce growth and toxin production by other strains of the same species. Generally, intraspecific competition has stronger negative effects than interspecific competition because members of the same species are more likely to occupy the same ecological niche [94]. In laboratory media, the most important factors likely at play are declining nutrient availabilities and increasing build-up of waste compounds. The generally slower growth and reduced spore production of *A. flavus* NRRL 35739 on artificial media and peanut hosts fits into the hypothesis that poor biocontrol strains are less able to out-grow toxigenic strains [18,19].

While the transcriptomics data suggest reasons why strain NRRL 35739 is a poor biocontrol agent, there nevertheless were several drawbacks to the chosen experimental and analytical methods. The cell culture conditions were not uniform across the various published studies, and the magnitudes of RNA reads and transcript counts also were very different. Conversion of the FPKMs into relative FPKMs permitted fairer comparison. However, this and the high thresholds for identification of genes only with large differences in relative expression among strains likely resulted in type II errors. None of the studies had biological or experimental replicates, and without replicates, statistical testing could not be performed. RT-PCR results did not show the expected large differences in transcript accumulation of the six stress response genes in strains NRRL 3357, NRRL 6513, and NRRL 35739, which may indicate fault(s) with the bioinformatics analysis or the RT-PCR experiment. Alternatively, the relative differences did not necessarily translate into large differences in absolute RNA transcript accumulation, at least for NRRL 35739 compared to the tested aflatoxigenic strains. It is still unknown if the differences would be more apparent between NRRL 35739 and a strong biocontrol agent. Additionally, RT-PCR primers for AFLA_006960 appeared to yield a second smaller product for strains NRRL 6513 and NRRL 35739. It is possible that mRNAs in these strains were subjected to alternative splicing not present in NRRL 3357.

Nonetheless, this pilot study provides an opening into the study of how genetic factors and abiotic stress tolerance may influence competitive growth by non-aflatoxigenic strains of *A. flavus*. To the authors’ knowledge, there are no previously published studies on the genetics of weak aflatoxin biocontrol agents; most relevant studies focus on identifying strong biocontrol agents. It is of particular interest that NRRL 35739 is the only non-aflatoxigenic strain for which researchers note that the strain sometimes increases aflatoxin accumulation [26]. The findings reported here provide other scientists with a starting point to further investigate how tolerance to abiotic stresses may influence competitive growth by non-aflatoxigenic strains of *A. flavus*. NRRL 35739 serves as a useful control for research into the largely uninvestigated factors that influence aflatoxin production when different *A. flavus* strains are grown together and the general mechanisms by which biocontrol strategies mitigate mycotoxin production in agriculture.

## Figures and Tables

**Table 1:** Primers used for PCR and RT-PCR.

Target	Sequences (5′-3′)	Amplicon (bp)
*GAPDH*	F- CACCTACGAGGACATCAAGAAG R- GATCAGGTCAGTGGAGACAATG	104
AFLA_006960	F- CAGACCGACTACCTCAACGA R- GCCTTCCTCTTCCTTGGTCT	147
AFLA_019230	F- AGGGTGGTCTTGGAAAGGTC R- TCTGCTTAACCTTGCCCTCA	93
AFLA_022380	F- CAAGCGCAACACCACAATTC R- CACGTTCACCCTCGTAAACC	101
AFLA_037820	F- CATCAAGCATACCGCCCAAA R- GCTTGGTTAACGCCAGGTAG	122
AFLA_060260	F- GAGGGTGAGAGCAAGGAAGT R- AGGATGCCGTTCTTAAGGCT	152
AFLA_079830	F- CGGTGCTGATCTCCTCATCA R- ACAGAGCGTGAATGATACCAG	71

**Table 2:** General information about RNA-Seq reads from other *Aspergillus flavus* transcriptomics studies.

Strain	SRA Run Accession	No. Reads	Read Length	Growth Condition ^a^	Reference
AF70	SRR7962692	20,657,024	1 × 99 bp	50 mL PDB, 31 °C, 10 days	[41]
NRRL 18543	SRR7962690	22,495,368	1 × 99 bp	50 mL PDB, 31 °C, 10 days	[41]
NRRL 3357	SRR544871	13,919,842	2 × 115 bp	100 mL PDB, 30 °C, shaking 200 rpm	[42]
WRRL 1519	SRR5907168	36,768,097	1 × 150 bp	20 mL PDB, 28 °C, shaking 150 rpm, 48 h	[10]

^a^ As much information as is available from each respective published study is provided; PDB, potato dextrose broth. All cultures were grown in the dark.

**Table 3:** Mapping of *A. flavus* RNA-Seq reads.

Strain	Reads Aligned to AF70 Genome (%)	No. Genes Expressed ^a^	Reads Aligned to NRRL 3357 Genome (%)	No. Genes Expressed
AF70	98.04	11,932	76.81	11,730
NRRL 18543	97.86	11,324	54.55	11,511
NRRL 3357	65.98	11,708	65.79	12,180
NRRL 35739	84.24	12,512	78.54	12,911
WRRL 1519	95.32	11,941	89.84	12,291

^a^ Expressed genes were those with fragments per kilobase of transcript per million mapped reads (FPKMs) greater than 0.

**Table 4:** Genes with differential relative expression of interest in the *A. flavus* AF70 alignment.

Gene Name	Putative Description	NRRL 35739 Relative FPKM	Average Fold Difference
AFLA70_147g002360	alternative oxidase	27	202
AFLA70_21g004231	isocitrate lyase AcuD	35	64
AFLA70_263g001160	phosphoenolpyruvate carboxykinase AcuF	14	38
AFLA70_286g001550	chitin synthesis regulation RCR superfamily	16	40
AFLA70_30g004530	membrane associated proteins in eicosanoid and glutathione metabolism family protein	11	17
AFLA70_338g001370	4-carboxymuconolactone decarboxylase	33	407
AFLA70_502g000700	heat shock protein, Hsp20-like	12	619
AFLA70_52g003980	domain of unknown function superfamily DUF1857	15	39
AFLA70_535g000630	molecular chaperone Hsp70	15	10
AFLA70_560g000680	heat shock protein, Hsp20-like	100	280
AFLA70_570g000531	domain of unknown function superfamily DUF4436; metal-dependent hydrolase	14	59
AFLA70_6g007820	hypothetical protein AFLA70_6g007820	14	36
AFLA70_73g004291	mitochondrial hypoxia responsive domain protein	16	16
AFLA70_89g003151	CsbD-like general stress response protein	40	274

**Table 5:** Genes with differential relative expression of interest in the *A. flavus* NRRL 3357 alignment.

Gene Name	Putative Description	NRRL 35739 Relative FPKM	Average Fold Difference
AFLA_006100	domain of unknown function superfamily DUF4243	7	20
AFLA_019230	CsbD-like general stress response protein	28	143
AFLA_035070	alternative oxidase	16	113
AFLA_036370	phosphoenolpyruvate carboxykinase AcuF	9	17
AFLA_037820	heat shock protein Hsp20 or Hsp30-like	7	300
AFLA_050290	amidohydrolase family protein	6	36
AFLA_052400	isocitrate lyase AcuD	20	30
AFLA_057980	conserved hypothetical protein	12	16
AFLA_060260	heat shock protein Hsp20 or Hsp30	58	130
AFLA_097370	chitin synthesis regulation RCR superfamily	9	17
AFLA_097530	domain of unknown function superfamily DUF1857	9	19
AFLA_124980	4-carboxymuconolactone decarboxylase	24	166

**Table 6:** Putative descriptions of stress-related *A. flavus* AF70 and NRRL 3357 genes.

AF70 ID	Annotation ^a^	NRRL 3357 ID	Annotation
AFLA70_128g002760	heat shock protein	AFLA_005950	stress response RCI peptide
AFLA70_132g002240	stress responsive A/B barrel domain protein	**AFLA_006960**	**molecular chaperone and allergen Mod-E/Hsp90/Hsp1**
AFLA70_135g002041	ATP-dependent chaperone	AFLA_007740	heat shock protein Hsp20/Hsp26
**AFLA70_166g001980**	**molecular chaperone and allergen Mod-E/Hsp90/Hsp1**	AFLA_012200	Hsp70 chaperone (HscA)
AFLA70_211g002041	Ca-activated chloride channel; membrane stress response protein	**AFLA_019230**	**CsbD-like general stress response protein**
AFLA70_23g004591	Hsp70 chaperone BiP/Kar2	**AFLA_022380**	**molecular chaperone Hsp70**
AFLA70_264g001290	Hsp70 nucleotide exchange factor (Fes1)	AFLA_025980	Hsp90 co-chaperone Cdc37
AFLA70_26g004611	Hsp70 chaperone (BiP)	AFLA_026660	stress responsive A/B barrel domain protein
AFLA70_275g001630	Hsp40 co-chaperone Jid1	AFLA_031070	Hsp40 co-chaperone Jid1
AFLA70_29g004401	stress response RCI peptide	AFLA_035620	Hsp70 chaperone BiP/Kar2
AFLA70_2g008980	stress response RCI peptide	**AFLA_037820**	**heat shock protein Hsp20 or Hsp30-like**
AFLA70_302g001550	Hsp90 co-chaperone Cdc37	AFLA_043390	Hsp70 chaperone
AFLA70_312g001481	Hsp70 chaperone (HscA)	AFLA_044620	mitochondrial Hsp70 chaperone (Ssc70)
AFLA70_33g004191	stress responsive A/B barrel domain protein	AFLA_045750	GroEL_like type I chaperonin
AFLA70_367g000950	oxidative stress protein Svf1	AFLA_052860	chaperone/heat shock protein Hsp9/Hsp12
AFLA70_46g003420	mitochondrial Hsp70 chaperone (Ssc70)	AFLA_055220	Hsp70 nucleotide exchange factor (Fes1)
**AFLA70_502g000700**	**heat shock protein, Hsp20-like**	**AFLA_060260**	**heat shock protein Hsp20 or Hsp30**
**AFLA70_535g000630**	**molecular chaperone Hsp70**	AFLA_068370	ATP-dependent chaperone
**AFLA70_560g000680**	**heat shock protein, Hsp20-like**	AFLA_071010	heat shock protein (Sti1)
AFLA70_57g003661	domain of unknown function superfamily 3759	**AFLA_079830**	**stress response RCI peptide**
AFLA70_640g000430	heat shock protein Hsp20/Hsp26	AFLA_084380	Hsp70 family protein
AFLA70_71g003911	chaperone, heat shock protein Hsp9/12	AFLA_084460	heat shock protein Hsp98/Hsp104/ClpA
**AFLA70_793g000140**	**stress response RCI peptide**	AFLA_092900	oxidative stress protein Svf1
AFLA70_80g002640	ATP-dependent chaperone ClipB	AFLA_095590	Hsp90 binding co-chaperone (Sba1)
AFLA70_83g002970	Hsp70 family protein	AFLA_095970	Hsp70 chaperone
**AFLA70_89g003151**	**CsbD-like general stress response protein**	AFLA_117640	domain of unknown function superfamily 3759
AFLA70_9g006590	heat shock protein, Hsp20-like	AFLA_119610	stress response RCI peptide

^a^ Bolded genes were observed to have higher relative expression in NRRL 35739 by the RNA-Seq data.

## Supplementary Materials

The following are available online at https://www.mdpi.com/2309-608X/5/2/53/s1. Figure S1. Relative gene expression based on read alignment to NRRL 3357. FPKM values were normalized to a 0 to 100 scale. Images were grouped by chromosomal arm.
