# Supplementary figures and images for: Aspergillus flavus NRRL 35739, a Poor Biocontrol Agent, May Have Increased Relative Expression of Stress Response Genes
Source: J Fungi (Basel). 2019 Jun 20;5(2):53. doi: 10.3390/jof5020053 (PMC6616650; doi:10.3390/jof5020053)

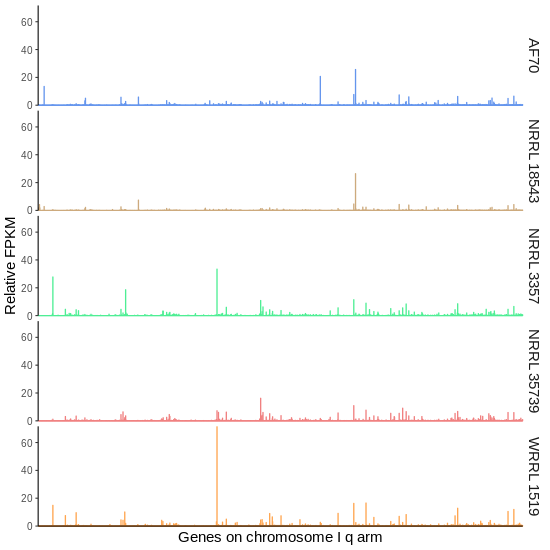

Supplement: Supplementary file 1 [file jof-05-00053-s001.zip › supplementary/SFig1A.tiff]

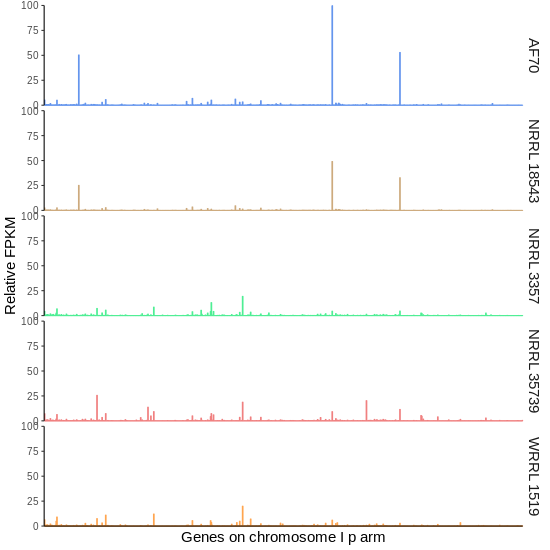

Supplement: Supplementary file 1 [file jof-05-00053-s001.zip › supplementary/SFig1B.tiff]

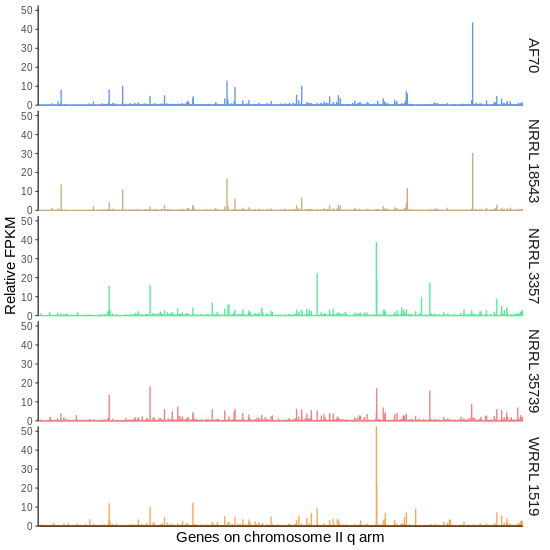

Supplement: Supplementary file 1 [file jof-05-00053-s001.zip › supplementary/SFig1C.tiff]

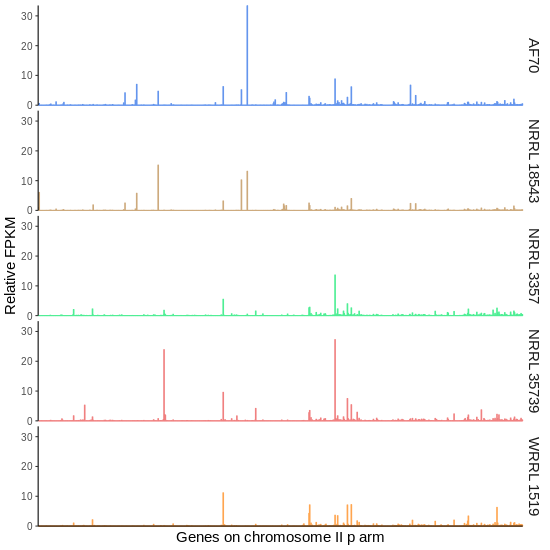

Supplement: Supplementary file 1 [file jof-05-00053-s001.zip › supplementary/SFig1D.tiff]

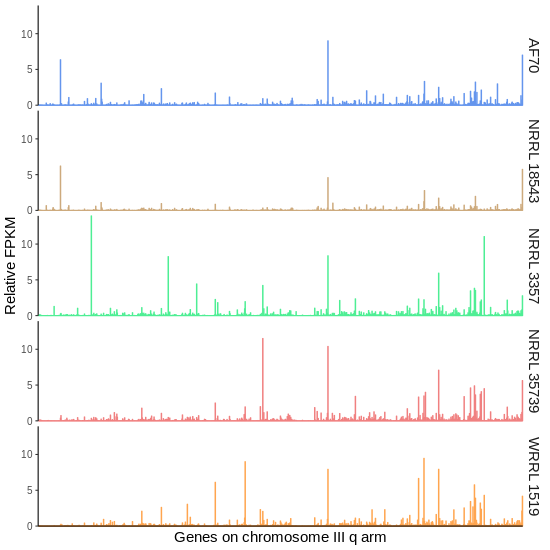

Supplement: Supplementary file 1 [file jof-05-00053-s001.zip › supplementary/SFig1E.tiff]

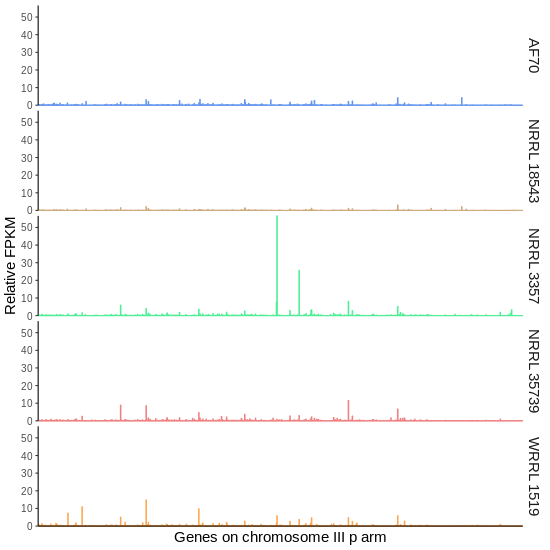

Supplement: Supplementary file 1 [file jof-05-00053-s001.zip › supplementary/SFig1F.tiff]

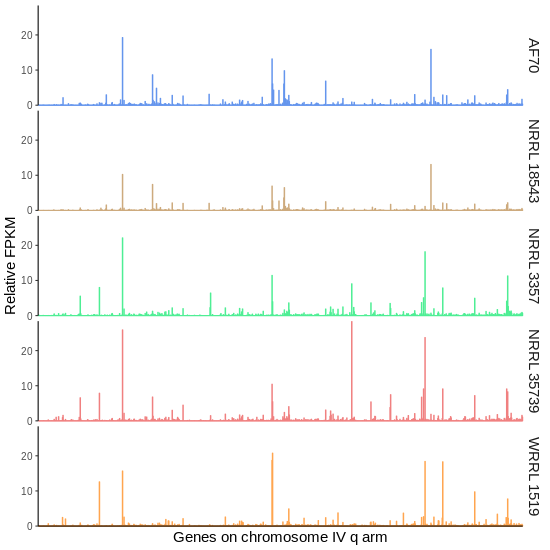

Supplement: Supplementary file 1 [file jof-05-00053-s001.zip › supplementary/SFig1G.tiff]

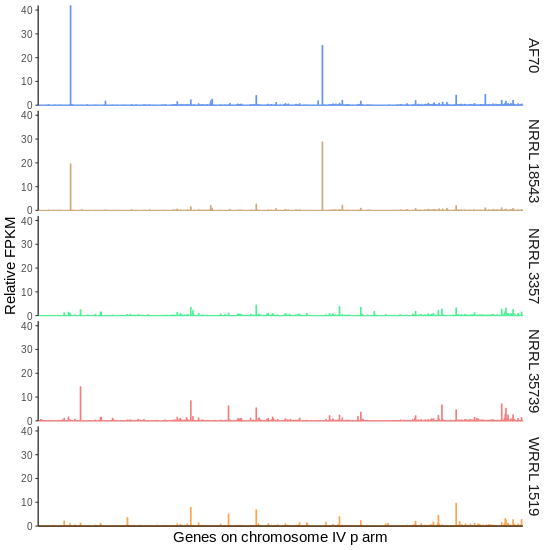

Supplement: Supplementary file 1 [file jof-05-00053-s001.zip › supplementary/SFig1H.tiff]

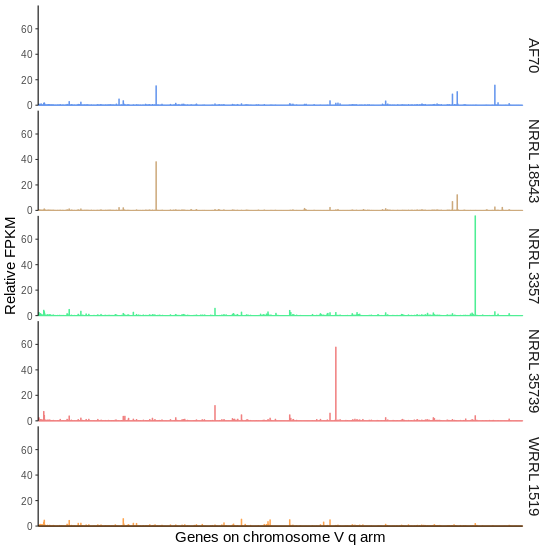

Supplement: Supplementary file 1 [file jof-05-00053-s001.zip › supplementary/SFig1I.tiff]

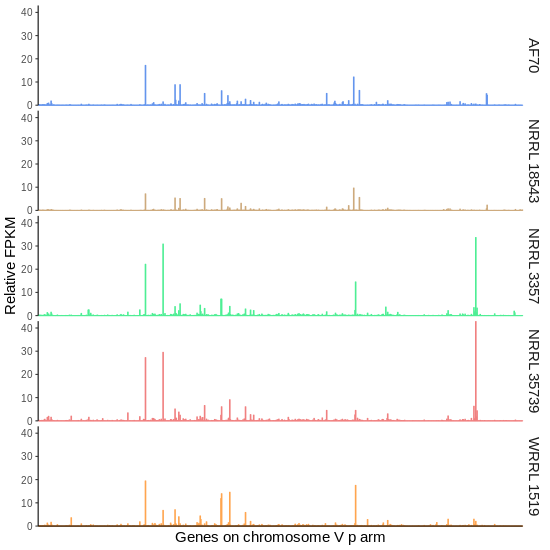

Supplement: Supplementary file 1 [file jof-05-00053-s001.zip › supplementary/SFig1J.tiff]

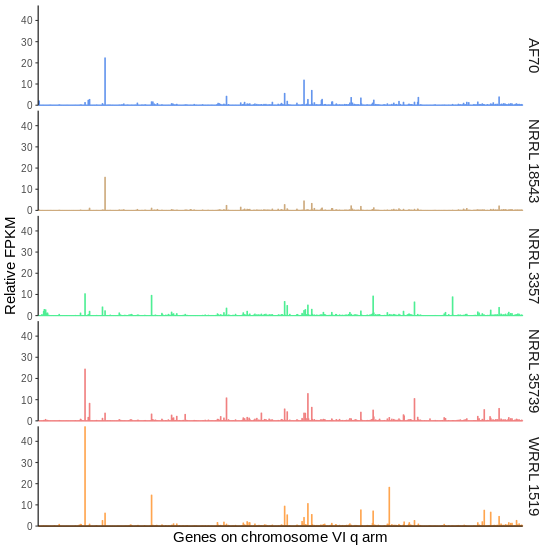

Supplement: Supplementary file 1 [file jof-05-00053-s001.zip › supplementary/SFig1K.tiff]

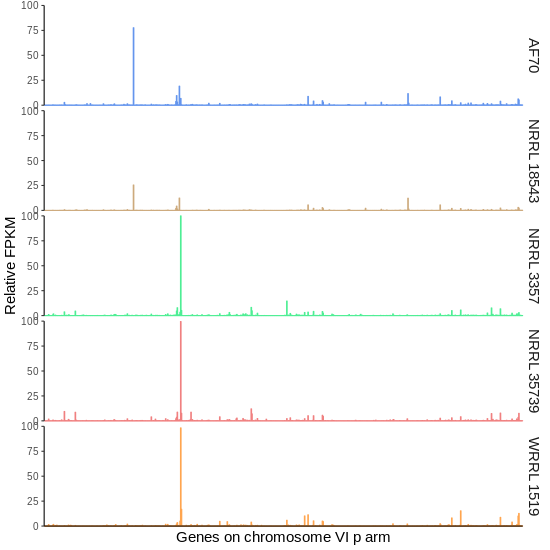

Supplement: Supplementary file 1 [file jof-05-00053-s001.zip › supplementary/SFig1L.tiff]

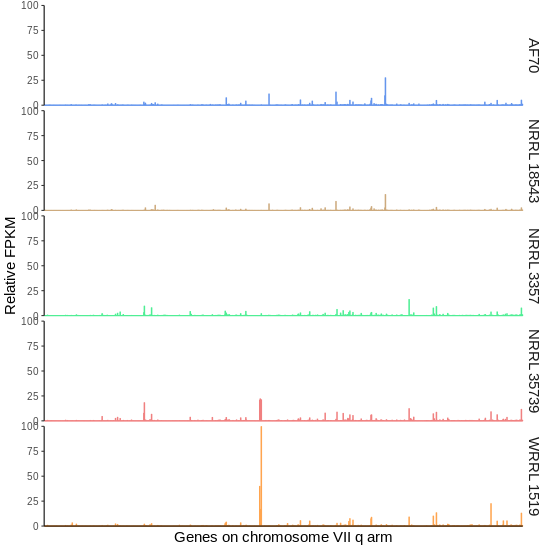

Supplement: Supplementary file 1 [file jof-05-00053-s001.zip › supplementary/SFig1M.tiff]

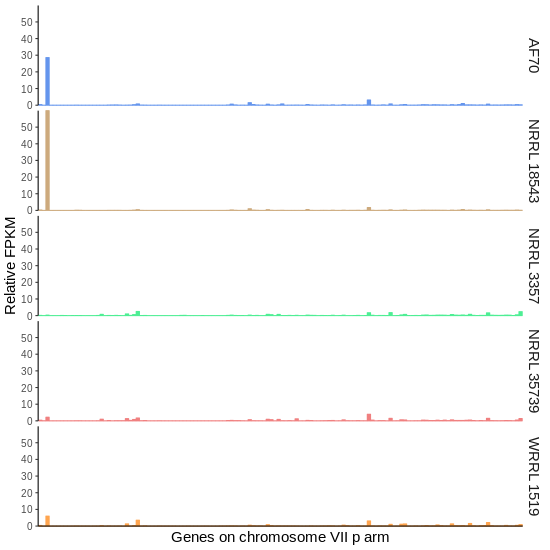

Supplement: Supplementary file 1 [file jof-05-00053-s001.zip › supplementary/SFig1N.tiff]

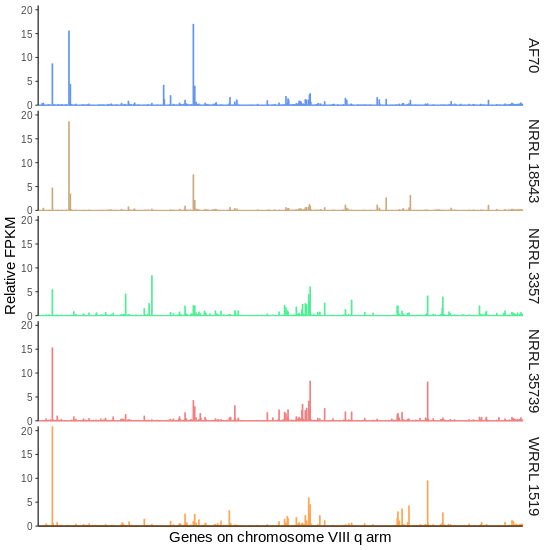

Supplement: Supplementary file 1 [file jof-05-00053-s001.zip › supplementary/SFig1O.tiff]

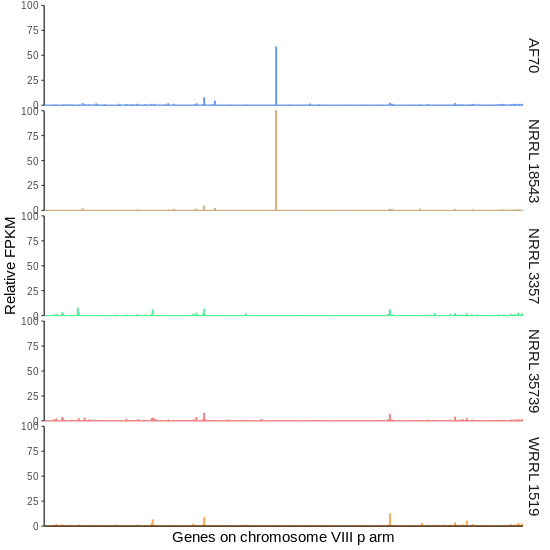

Supplement: Supplementary file 1 [file jof-05-00053-s001.zip › supplementary/SFig1P.tiff]
